# Rationale and study design of the Japan environment and children’s study (JECS)

**DOI:** 10.1186/1471-2458-14-25

**Published:** 2014-01-10

**Authors:** Toshihiro Kawamoto, Hiroshi Nitta, Katsuyuki Murata, Eisaku Toda, Naoya Tsukamoto, Manabu Hasegawa, Zentaro Yamagata, Fujio Kayama, Reiko Kishi, Yukihiro Ohya, Hirohisa Saito, Haruhiko Sago, Makiko Okuyama, Tsutomu Ogata, Susumu Yokoya, Yuji Koresawa, Yasuyuki Shibata, Shoji Nakayama, Takehiro Michikawa, Ayano Takeuchi, Hiroshi Satoh

**Affiliations:** 1 National Center for Japan Environment and Children’s Study, National Institute for Environmental Studies, 16-2, Onogawa, Tsukuba 305-8506, Japan; 2 Department of Environmental Health, University of Occupational and Environmental Health, 1-1 Iseigaoka, Yahatanishi-ku, Kitakyushu 807-8555, Japan; 3 Department of Environmental Health Sciences, Akita University Graduate School of Medicine, 1-1 Hondo, Akita City 010-8543, Japan; 4 Environment Risk Assessment Office, Environmental Health Department, the Ministry of the Environment, 1-2-2 Kasumigaseki, Chiyoda-ku, Tokyo 100-8975, Japan; 5 Department of Health Sciences, Interdisciplinary Graduate School of Medicine and Engineering, University of Yamanashi, 1110 Shimogato, Chuo, Yamanashi 409-3898, Japan; 6 Division of Environmental Toxicology, School of Medicine, Jichi Medical School, 3311-1 Yakushiji, Shimotsuke-City, Tochigi 329-0498, Japan; 7 Hokkaido Unit Center, Center for Environmental and Health Sciences, Hokkaido University, Kita 12, Nishi 7, Kita-ku, Sapporo-City, Hokkaido 060-0812, Japan; 8 Medical Support Center for Japan Environment and Children’s Study, National Center for Child Health and Development, 2-10-1 Okura, Setagaya-ku, Tokyo 157-8535, Japan

**Keywords:** Birth cohort, Children, Growth and development, Environmental chemicals, Pregnant women

## Abstract

**Background:**

There is global concern over significant threats from a wide variety of environmental hazards to which children face. Large-scale and long-term birth cohort studies are needed for better environmental management based on sound science. The primary objective of the Japan Environment and Children’s Study (JECS), a nation-wide birth cohort study that started its recruitment in January 2011, is to elucidate environmental factors that affect children’s health and development.

**Methods/Design:**

Approximately 100,000 expecting mothers who live in designated study areas will be recruited over a 3-year period from January 2011. Participating children will be followed until they reach 13 years of age. Exposure to environmental factors will be assessed by chemical analyses of bio-specimens (blood, cord blood, urine, breast milk, and hair), household environment measurements, and computational simulations using monitoring data (e.g. ambient air quality monitoring) as well as questionnaires. JECS’ priority outcomes include reproduction/pregnancy complications, congenital anomalies, neuropsychiatric disorders, immune system disorders, and metabolic/endocrine system disorders. Genetic factors, socioeconomic status, and lifestyle factors will also be examined as covariates and potential confounders. To maximize representativeness, we adopted provider-mediated community-based recruitment.

**Discussion:**

Through JECS, chemical substances to which children are exposed during the fetal stage or early childhood will be identified. The JECS results will be translated to better risk assessment and management to provide healthy environment for next generations.

## Background

The Japan Environment and Children’s Study (JECS) is a nation-wide and government funded birth cohort study that started recruiting expecting mothers in January 2011. JECS is aimed to provide the foundation for policy making to safeguard the environment for the next generations.

The Miami Declaration on Children’s Environmental Health was adopted at the G8 Environment Ministers’ Meeting held in Miami in 1997, in the midst of a growing concern regarding the effects that environmental pollution posed to children, and the acknowledgement of the vulnerability of children to harmful substances in the environment. The World Health Organization (WHO) published a report in 2006 in which approximately 40% of all children’s death was attributed the environment [1]. In order to address these concerns, Denmark and Norway commenced large-scale epidemiological studies with approximately 100,000 participants targeting children in late 1990’s [2,3]. The United States is preparing a similar study, the National Children’s Study [4]. The importance of children’s environmental health was highlighted again at the G8 Environment Ministers’ Meeting held in Syracuse, Italy, in 2009, where ministers agreed to cooperate in scientific research to push this movement forward.

In Japan, the Advisory Board on Children’s Environmental Health, established by the Ministry of the Environment (MOE), proposed a large-scale birth cohort study in order to evaluate the effects of environmental chemicals on children’s health and development. In April 2008, the Working Group of the Epidemiological Research for Children’s Environmental Health (later JECS Working Group) was organized and started systematic reviews on existing epidemiological findings regarding health impact of chemical exposures and the roles of potential confounders and effect modifiers, such as other environmental factors, genetic factors, socioeconomic status and lifestyle, in order to develop JECS study design and hypotheses. In March 2010, JECS Working Group published a draft conceptual plan for a large-scale birth cohort study covering all of Japan [5]. The budget for conducting JECS was approved by the Diet in 2010 and JECS was launched in April 2010.

The ultimate goal of JECS is “to identify environmental factors that affect children’s health and development in order to help decision makers design better chemical risk management strategies”.

## Methods/Design

### Implementation structure

**Figure 1:**
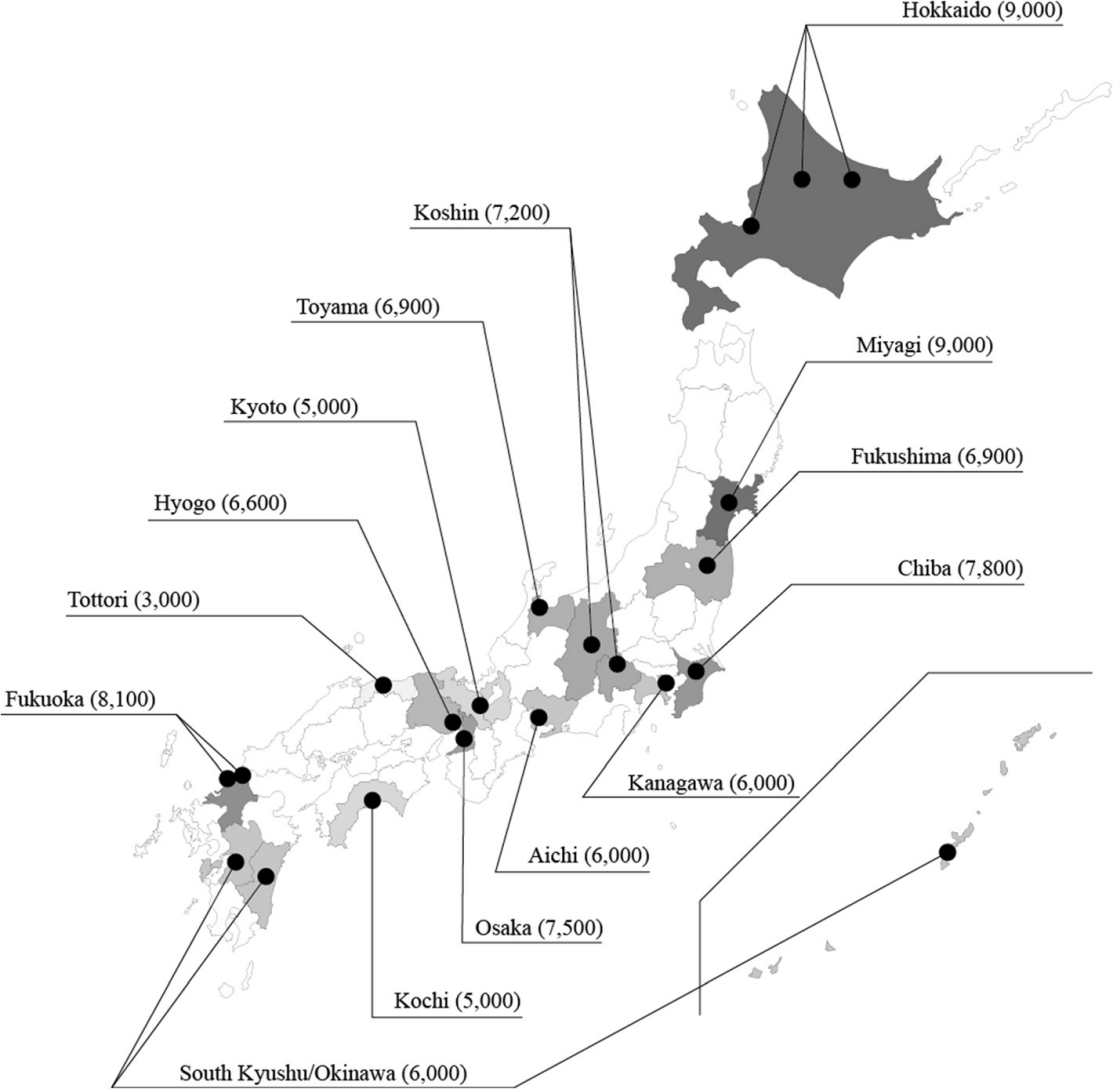
Study areas and targeted sampling sizes of JECS as of January 2011.

One of the major characteristics of JECS is that it is implemented as a national project funded directly by MOE, in contrast with most of the other epidemiological studies that are carried out in Japan by universities or research institutes using government research subsidies. The JECS is structured by three-tier centers. The National Center for JECS (program office), established in the National Institute for Environmental Studies (NIES), leads the JECS, while the National Center for Child Health and Development (NCCHD) supports the National Center as the Medical Support Center with its medical expertise. The National Center and Medical Support Center will cooperate together with 15 Regional Centers, located from the north, Hokkaido, to the south, Okinawa (Figure 1). The Regional Centers, which are seated in universities, are responsible for recruiting study participants and conducting follow-up programs in respective study areas, collaborating with local governments. Local health care providers including hospitals and clinics within or in the vicinity of study areas, agreed to participate in JECS in response to the Regional Centers’ requests for cooperation. The recruitment and bio-specimen collections are performed in the above mentioned “cooperating health care providers” under the control of the Regional Centers.

The JECS protocol was approved by the Institutional Review Board (IRB) on epidemiologica studies of MOE, and Ethics Committees of all participating institutions, i.e., NIES, NCCHD, Hokkaido University, Sapporo Medical University, Asahikawa Medical College, Japanese Red Cross Hokkaido College of Nursing, Tohoku University, Fukushima Medical University, Chiba University, Yokohama City Unversity, University of Yamanashi, Shinshu University, Univresity of Toyama, Nagoya City University, Kyoto University, Doshisha University, Osaka University, Osaka Medical Center and Research Institute for Maternal and Child Health, Hyogo College of Medicine, Tottori Unversity, Kochi Univeristy, University of Occupational and Environmenatal University, Kyushu University, Kumamoto University, University of Miyazaki, and University of the Ryukyus. The JECS will be conducted in acoordance to the Helsinki Declaration and to the other nationally valid regulations.

### Study areas

To ensure generalizability and ability to extrapolate the results of JECS to Japanese population, the 15 Regional Centers are selected to cover wide geographical areas. The study locations’ urbanization and land development are diverse, from urban and suburban to rural areas as well as from agricultural and fishery to commercial and industrial uses.

Regional Centers were selected in a competitive process in which universities and other research institutions were invited to submit proposals for covered areas and population, recruitment methods, organization structures, regional liaison, and the resources. Each Regional Center consists of one or more study areas. The population of the selected study areas is 130,000 to 600,000. Assuming birth rate of the study areas to be 1%, each Regional Center will see 1,300 to 6,000 annual births, 4,400 on average. JECS aims half of all the births in the area to be covered. Selected Regional Centers are required to recruit 3,000 to 9,000 pregnant women in three years, totaling to 100,000 participants in 15 Regional Centers (Figure 1). In order to ensure the maximum contact with eligible participants, Regional Centers have formed JECS regional liaison involving local governments and health care providers. All the study areas are contained within administrative units, e.g. municipalities, further enhancing local government cooperation. This makes it easier to obtain basic health statistics in the study areas, for example, total number of births, sex ratios, birth weights, morbidities, and mortalities. It also helps us maximize follow-up and retention rates.

### Study subjects

Hundred thousand is the targeted number of enrollment of pregnant women. The recruitment period started in January 2011 and will continue for three years until March 2014. In Japan, 1,070,025 babies were born in 2009. One hundred thousand per three years (i.e. 33,333 per year) is around 3% of Japanese newborns. Partners are also recruited but their participation is not mandatory.

The eligibility criteria for participants (expecting mothers) are as follows: 1) They should reside in the study areas at the time of the recruitment, and are expected to reside continually in Japan for the foreseeable future, 2) expected delivery date should be between 1 August 2011 and mid-2014, and 3) they should be capable to participate in the study without difficulty, i.e., must be able to comprehend the Japanese language and complete the self-administered questionnaire. Those residing outside the study areas, even if they visit the cooperating health care providers within the study areas, are excluded from the study.

### Recruitment strategies

**Figure 2:**
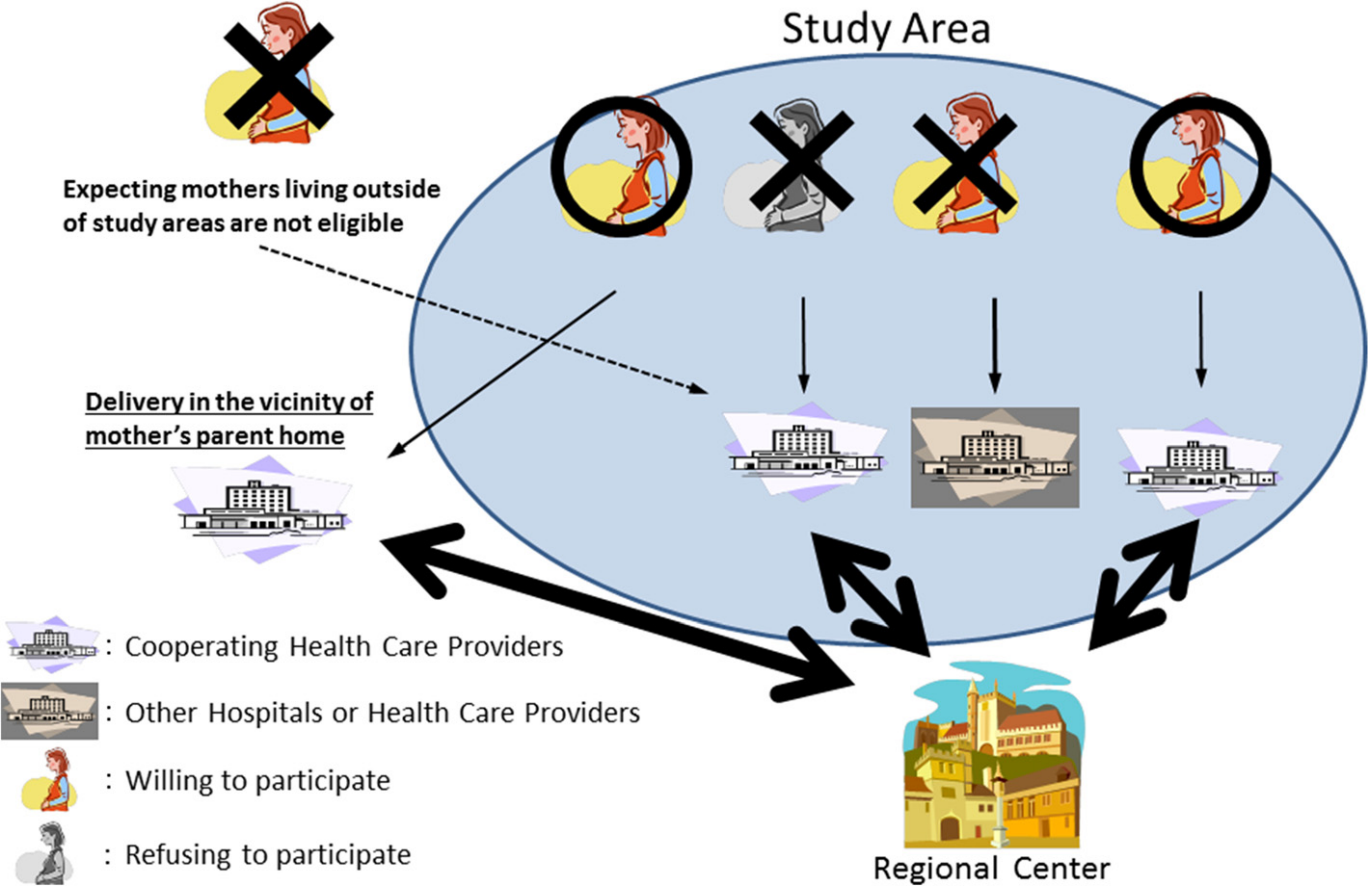
**Provider-mediated community-based recruitment.** Regional Center requests all obstetric facilities which expecting mothers residing within study areas are thought to visit for prenatal exams and childbirth to cooperate with JECS. Obstetric facilities confirming cooperating in JECS will be designated as cooperating health care providers, and invite all expecting mothers residing in the study areas who visit there to participate in JECS. In order to achieve a coverage ratio in excess of 50%, for example, the Regional Center shall exert its best efforts to receive cooperation from obstetric facilities to cover over 70% of all births in the study areas and the respective cooperating health care providers (cooperating obstetric facilities) shall recruit over 70% of eligible expecting mothers at their first prenatal examination.

We make contact with as many expecting mothers who reside in study areas as possible. The recruitment rate is targeted to be more than 50% of all eligible mothers. Either or both of the following two recruitment protocols are applied: 1) recruitment at the time of first prenatal examination at cooperating health care providers, i.e. obstetric facilities (provider-mediated community-based recruitment; Figure 2), and/or 2) recruitment at local government offices issuing pregnancy journals, namely Mother-Child Health Handbooks (the Mother-Child Health Handbook is an official booklet which all expecting mothers in Japan are given complimentary when they become pregnant in order to receive municipal services for pregnancy, delivery, and childcare).

Written informed consent for participation in the study is obtained from individual mothers and their partners, and for children from their parent or guardian. The participants are able to withdraw from the study at any time. Excluded are those who do not consent to the study protocol and cannot be accessed during the pregnancy period. In Japan, it is not uncommon that mothers return to their parents’ home to give birth. Those who plan to move back to parents’ home are not eligible except they are accessible by the Regional Centers.

Though we carefully planned the sampling, the recruitment is not completely random. The recruitment activities are taking place at health care providers as well as local government facilities. We make every effort to reach out as many eligible women in the study areas as possible. The representativeness of the JECS samples will be evaluated when the birth data are fixed.

### Follow-up programs

**Figure 3:**
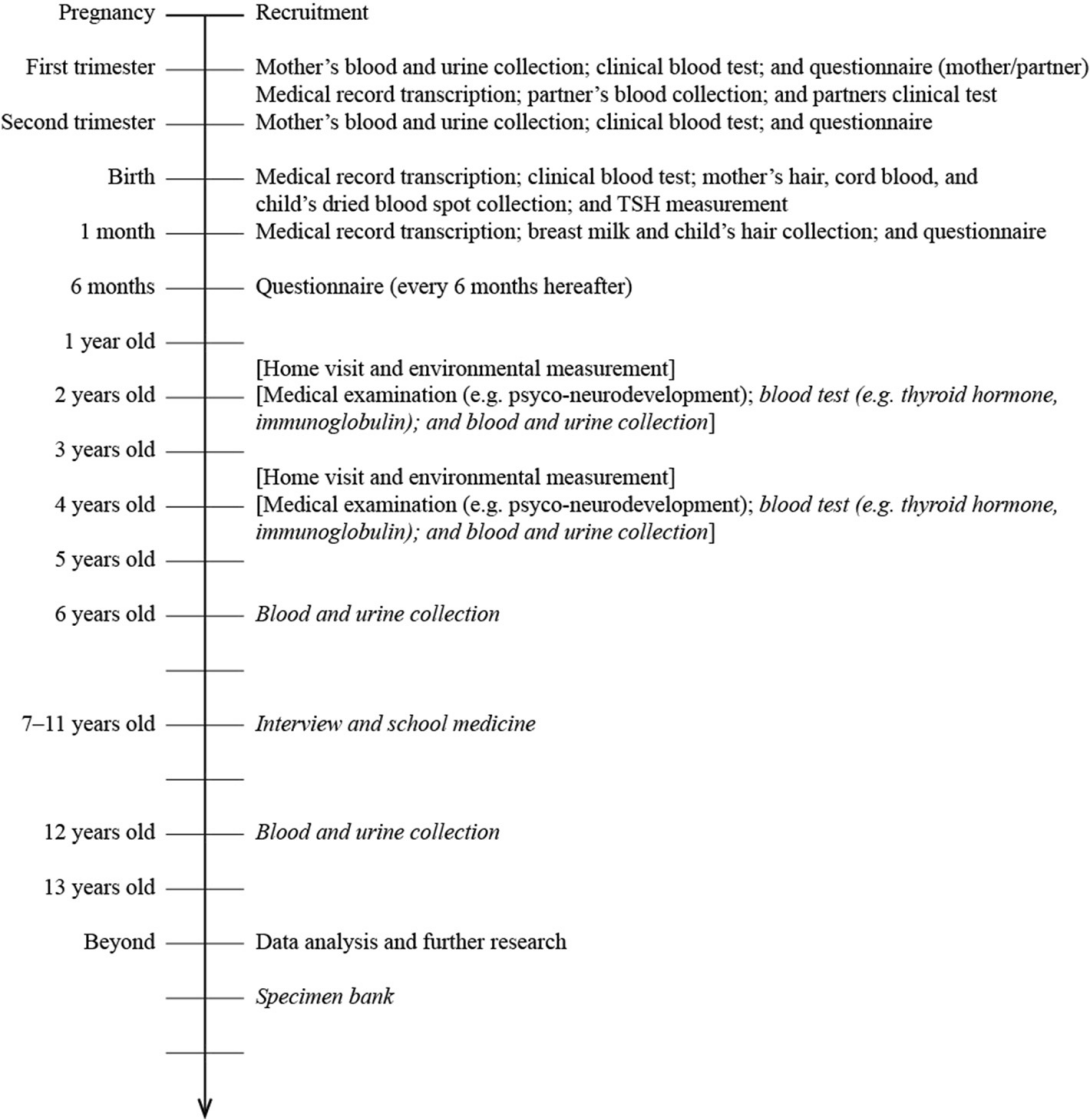
**Follow-up programs.** Items in brackets are performed on the sub-cohort and ones in italic face are plans under discussion.

The participating children are followed up until they reach the age of 13 years (Figure 3). The follow-up are carried out mainly by self-administered questionnaire. We recruit eligible women as in early pregnancy as possible. Questionnaires are administered to enrolled mothers and their partners at first trimester and second/third trimester in pregnancy. Medical histories of past and present pregnancies are recorded. Whole blood, plasma, and urine samples are collected from mothers twice during pregnancy. Whole blood and plasma are collected once from their partners. At birth, maternal blood and hair, cord blood, and baby’s dried blood samples are obtained. A month after birth, a questionnaire is answered by the mothers/partners; mothers’ and children’s medical records are copied; and breast milk and babies’ hair are sampled. Thereafter, questionnaires are sent out every 6 months.

As shown in Figure 3, blood and urine collection at the age of 2 and 4 years from selected children of a sub-cohort and at 6 and 12 years from all participating children is under discussion. We have not received the approval of the phlebotomy on children from IRB of MOE or any Ethics Committees of participating institutions at present. In case that invasive tests including the phlebotomy are determined to be conducted on children, we will obtain a new approval from the IRB and the all Ethics Committees, then written consents from parents/guardians of the target children before any of the invasive procedures take place. The participants are able to withdraw from the study at any time. They can also reject the invasive tests but remain in the study.

When participants become insensitive during the course of the follow-up program, we give them telephone calls to encourage them to come back and/or access to the register of residents in municipal offices to investigate whether they have moved out the study areas. When participants move out of study areas but still accessible, the follow-up programs, including the questionnaires and other information collection, will be continued. Eighty percent retention is targeted.

### Outcome measurements

Five priority outcomes are: 1) reproduction and pregnancy complications (e.g. abnormal pregnancies, premature birth, unbalanced sex ratio, and miscarriage), 2) congenital anomalies (ventricular septal defects, hypospadias, undescended testis, cleft lip/cleft palate, and chromosomal anomalies), 3) neuropsychiatric disorders (autism spectrum disorders, learning disorders, and attention-deficit hyperactivity disorder), 4) allergies and immune system deficiencies (asthma, atopic dermatitis, and food allergies), 5) metabolism and endocrine system disorders (impaired glucose tolerance, obesity, impact on reproductive organs, impaired genital formations, and sexual differentiation disorder). However, hundred thousand is not enough to analyze the association between environmental exposures and cancers. JECS collects cancer information in order to contribute future international pooled analysis, e.g. International Childhood Cancer Cohort Consortium (I4C) [6].

From the JECS cohort, a sub-cohort with the size of 5,000 will be extracted. In that sub-cohort extended outcome measurements are planned, for instance, clinical analysis of blood samples from children; face to face interviews by medical staffs to evaluate neurological development; and medical examination.

### Exposure assessment

Environmental factors are manifold and complicated. In order to evaluate exposure to a wide range of environmental factors, the following four approaches are employed:

1. Questionnaires

A part of each questionnaire is designated to collect information about chemical exposure, e.g. the use of organic solvents, kerosene, pesticides, disinfectants, heavy metals, antineoplastic drugs, narcotics, paints, hair dyes, and printer inks. Exposure to noise, vibration, high/low temperature, and dusts is also asked in the questionnaires.

2. Chemical analysis of bio-specimens

Chemical substances or their metabolites are measured in peripheral blood, cord blood, breast milk, urine, and hair. Target compounds are shown in Table 1.

3. Environmental measurements

In the same sub-cohort as the one described above, indoor air pollutants, including volatile organic compounds (VOCs), aldehydes, nitrogen oxides, and fine particulate matters (PM_2.5_), will be measured during home visits. Noise levels and other physical parameters such as temperature and humidity will also be assessed.

4. Atmospheric simulation from ambient air quality monitoring

There are about 1,500 ambient air quality monitoring stations and about 500 roadside air quality monitoring stations across Japan, where levels of the five classical air pollutants, i.e., carbon monoxide (CO), suspended particulate matter (SPM), sulfur dioxide (SO_2_), nitrogen dioxide (NO_2_), and photochemical oxidants are monitored continuously. Twenty other hazardous air pollutants are also monitored at over 300 sites. Exposure to classical and hazardous air pollutants will be estimated from the monitoring station data using atmospheric simulation models.

**Table 1:** Target compounds to be analyzed in bio-specimens

**Group**	**Target compounds**
Metals	Lead, cadmium, total mercury, methyl mercury, arsenics and its compounds including, arsenobetaine, metylarsonic acid, dimethylarsinic acid, trimethylarsine oxide, etc.
Inorganic substances	Iodine, perchlorate, nitrate nitrogen, etc
Chlorinated POPs (Persistent organic pollutants)	Polychlorinated biphenysl (PCBs), hydroxylated polychlorinated biphenyl (OH-PCB), dioxins (PCDDs, PCDFs, Co-PCBs), pexachlorobenzene (HCB), pentachlorobennzene (PeCB), etc.
Pesticides (including pesticide-POPs)	Chlordanes, DDT and its metabolites (DDE, etc.), drin compounds for agriculture (dieldrin, etc.), heptachlor, hexachlorocyclohexaxne (HCH), mirex, chlordecone, toxaphene, organophophorus pesticide metabolites (DMP, DEP, DMTP, DETP, etc.), fenitrothion metabolite (methylnitrophenol), acephate metabolite (methamidophos), pyrethroid metabolites (PBA, DCCA, etc.), dithiocarbamate fungicide metabolites (ethylene thiourea, etc.), neonicotinoid metabolites, pentachlorophenol (PCP), atrazine, dymron, glyphosate, flutolanil, iprodione, flusulfamide, etc.
Brominated POPs	Polybromodiphenylethers (PBDEs), polybromobiphenyls (PBBs), hexabromocyclododecan (HBCD), etc.
Organofluorine compounds	Perfluorooctanoic acid (PFOA), perfluorooctane sulfonate (PFOS), perfluorononanoic acid (PFNA), etc.
Aroma compounds	Nitromusks, cyclic musks, etc.
Phthalate metabolites	Mono (2-ethylhexyl) phthalates, etc.
Phenols	Bisphenol A, Nonyphenols, Parabens, etc.
Others	Triclosan, benzophenone, N, N-diethyl-meta-toluamide (DEET), polyaromatic hydrocarbons (PAHs) and their metabolites (1-hydroxypyrene, 3-hydroxyphenanthrebe, etc.), cotinine, thiocyanate, dichlorobenzene, phytoestrogen, caffeine, pyridine, acrylamide, tributyl phosphate, tributoxyethl phosphate, 8-hydroxydeoxyguanosine (8-OHdG), etc.

### Genetic analyses

Blood samples are collected from mothers, their partners, and children. The samples are stored at the National Center for genetic analyses. Single mutation and/or genome wide analyses are planned.

### Covariates and potential confounders

Other covariates or potential confounders measured in JECS include socioeconomic status (e.g. education, employment, house-hold income, social capital, and community support), lifestyle factors (stress levels, diet, smoking and alcohol habits, physical exercise activities, sleep, infections, and medications), and physical environment (heat, ionizing radiation, housing condition, and neighborhood). Biochemical tests (e.g. immunoglobulin E, glycated hemoglobin, and cholesterol) are performed on maternal, partners’, and cord blood samples. Thyroid-stimulating hormone is analyzed for in dried blood spots from new born babies.

### Data management system and Bio-specimen storage

All data collected from the participants are maintained by a data management system (DMS) developed by the National Center. Questionnaires are scanned and transformed to electronic data by optical character recognition (OCR) at the Regional Centers, then transferred to the DMS. Clinical chemistry data are electronically sent to the National Center and loaded on to the DMS. Personally identifiable information is stored separately from the data. All access to the DMS is recorded.

Bio-specimens including blood, cord blood, urine, breast milk, and hair provided by the participating mothers, partners and their children are archived in three different storage facilities located in the National Center and elsewhere. Samples waiting for chemical analysis are stored in negative 80 degrees Celsius. Some aliquots of samples are reserved in liquid nitrogen tanks for much longer period for future analysis. A computer assisted repository system was developed in order to securely manage the bio-specimens for long-term. Chemical analysis will start after completion of the recruitment in early 2014.

At this point, the JECS study is planned to continue until 2032, five years after all the participant children reach 13 years of age, allowing thorough data analysis. JECS may be extended beyond 2032 to further examine adolescence’s health. The bio-specimen repository may be converted to a bio-bank upon completion of the JECS study to contribute further scientific research. All of these possibilities are written in the consent form.

### Adjunct studies

In addition to the JECS main study, adjunct studies are conducted by the National Center, Medical Support Center, Regional Centers, or any combination of them using their own funding. The adjunct studies may include procedures that are not adopted by the main study, e.g. collection and examination of placenta. Proposals for adjunct studies need to be approved by MOE, ensuring that they do not interfere with the main study.

## Discussion

In recent years, there has been a growing concern regarding the vulnerability of children to harmful substances in the surrounding environment. Children are still undergoing development and the structure and function in each organ reach maturity at differing stages. Exposure to toxic chemicals during certain periods of development, namely critical windows, may lead to much severer consequences than the similar exposure in adulthood [4].

The environment surrounding us has become quite different from what it used to be. The floors and walls of our houses are made of new materials. Even though floors are usually made from wood, their coatings which children touch may not be natural but artificial polymers. Our clothes have also changed from cotton and silk to polyester and acrylic fibers. Toys are no longer made from bamboo and wood but from plastics. The interior of residential houses, offices, and vehicles has also changed from traditional materials to industrial ones. Fire regulation now requires thermoplastics, thermosets, textiles and coatings be treated with flame retardants. Children eat processed foods, wrapped in plastics, and are living in the environment filled with novel chemicals. Even though those chemicals are thoroughly tested before they reach the market, we have little knowledge about their effects on children’s health and development, especially when they exist as a mixture. The effect of those chemicals could be subtle. When one wants to examine not only such minute association of the chemicals with children’s health but also the causality, a large scale prospective study is the only solution. JECS is an extremely ambitious project, run by the Japanese government, which is aimed to evaluate the impact of the environment in which our children live on their health and development. The fruit of JECS will therefore be with no doubt translated into better regulations and policies.

Two birth-cohorts have been conducted in Japan, the Hokkaido Study of Environment and Children’s Health and Tohoku Study of Child Development, provided a good basis for developing the JECS design. The Hokkaido Study began in 2002 and its population consists of 20,000 children [7]. It started with similar aims to JECS but at a smaller scale. The Tohoku Study, which started in 2001, focuses on neurobehavioral development, subjecting approximately 600 children. What makes the Tohoku Study characteristic is their extensive chemical exposure evaluation as well as evaluation of children’s development utilizing face to face interviews and home visits [8,9]. The protocols of JECS were developed based on the experience of both studies, including their biomonitoring data.

The recruitment of JECS started in January 2011. Albeit Japan has suffered from severe damage caused by the mega-earth quake and tsunami, the number of participants increased steadily after March 2011. As of March 14, 2013, the number of enrollment reached 62,751 mothers and 28,982 partners. Mothers gave birth to 40,144 babies. Cord blood was collected form 38,008 new born babies. At the current recruitment rate, we expect to enroll 100,000 participants by early 2014 as planned.

The budgets which had been spent to conduct JECS were 2.5, 4.6, and 6.1 billion yen (25, 46, and 61 million US dollars) in 2010, 2011, and 2012 Japanese fiscal years (starting April), respectively.

Chemical analysis of bio-specimens and data analysis will start shortly after the completion of the recruitment in early 2014. The first publication that reports the association between environmental factors and some early stage outcomes such as pregnancy complications, congenital anomalies, and birth data will become available within the next few years.

## Abbreviations

CO: Carbon monoxide; DMS: Data management system; I4C: International Childhood Cancer Cohort Consortium; IRB: Institutional review board; JECS: Japan Environment and Children’s Study; MOE: The Ministry of the Environment; NCCHD: National Center for Child Health and Development; NIES: National Institute for Environmental Studies; NO2: Nitrogen dioxide; OCR: Optical character recognition; PM: Fine particulate matter; SO2: Sulfur dioxide; SPM: Suspended particulate matter; VOC: Volatile organic compound; WHO: World Health Organization.

## Competing interests

All the authors of this manuscript have no competing interests as defined by BioMed; we declare that we do not have any other interests that influence the results and discussion of this paper.

## Authors’ contributions

The authors are justifiably credited with authorship, according to the authorship criteria. TK is primary investigator of this study. HiSat is ex-primary investigator. HN, KM, TK and HiSat conceived the study idea, designed the study. NT, ET, YK and MH carried out administrative procedure. RK, KM, FK and ZY supervised the study design and protocol development. YO, HiSai, HaS, TO, SY, MO and MT supervised the study from the point of medical and clinical aspect. YS and SN supervised the study from the point of exposure science. HN and AT contributed to develop the study protocol in the point of statistical view. TK, ET, SN, TM and AT wrote the draft and edited the manuscript. All authors participated in the discussion of the protocol development and revision of the manuscript. All authors critically revised the manuscript and approved the final version.

## Authors’ information

Toshihiro Kawamoto is the director of National Center for Japan Environment and Children’s Study (JECS), National Institute for Environmental Studies (NIES), and the professor of Department of Environmental Health, University of Occupational and Environmental Health.

Hiroshi Nitta is the acting director of National Center for JECS, and the director of Center for Environmental Health Sciences, NIES.

Katsuyuki Murata is the professor of Department of Environmental Health Sciences, Akita University Graduate School of Medicine.

Eisaku Toda is the former director of Environment Risk Assessment Office, Environmental Health Department, Ministry of the Environment (MOE) and now the director of International Strategy Division, Global Environment Bureau, MOE, 1-2-2 Kasumigaseki, Chiyoda-ku, Tokyo 100-8975, Japan.

Naoya Tsukamoto is the former director of Environment Risk Assessment Office, Environmental Health Department, MOE, and now the director of Industrial Waste Management Division, MOE, 1-2-2 Kasumigaseki, Chiyoda-ku, Tokyo 100-8975, Japan.

Manabu Hasegawa is the former deputy director of Environment Risk Assessment Office, Environmental Health Department, MOE, and now a deputy director of Guidance of Medical Service Division, Health Policy Bureau, the Ministry of Health, Labour and Welfares, 1-2-2, Kasumigaseki, Chiyoda-ku, Tokyo 100-8916, Japan.

Zentaro Yamagata is the professor of Department of Health Sciences, Interdisciplinary Graduate School of medicine and Engineering, University of Yamanashi.

Fujio Kayama is the professor of Division of Environmental Toxicology, School of Medicine, Jichi Medical University.

Reiko Kishi is the director of Hokkaido Unit Center for JECS, and a professor emeritus of Hokkaido University.

Yukihiro Ohya is the acting director of Medical Support Center for JECS, National Center for Child Health and Development (NCCHD).

Hirohisa Saito is the director of Medical Support Center for JECS and also the deputy director of National Research Institute for Child Health and Development, NCCHD.

Haruhiko Sago, Makiko Okuyama and Susumu Yokoya are directors of NCCHD and also belong to Medical Support Center for JECS, NCCHD.

Tsutomu Ogata was a director of NCCHD and is now the professor of Department of Pediatrics, Hamamatsu University School of Medicine, 1-20-1 Handayama. Higashi-ku, Hamamatsu-city, Shizuoka 431-3192, Japan.

Yuji Koresawa is the former deputy director of National Center for JECS and is now the director of Office of Waste Disposal Management, MOE. 1-2-2, Kasumigaseki, Chiyoda-ku, Tokyo 100-8975, Japan.

Yasuyuki Shibata is a researcher of National Center for JECS and senior scientist of NIES.

Shoji Nakayama is a senior scientist of National Center for JECS and a chief of Center for Environmental Health Sciences, NIES.

Takehiro Michikawa and Ayano Takeuchi are researchers of National Center for JECS and Center for Environmental Health Sciences, NIES

Hiroshi Satoh is the former director of National Center for JECS and now an acting director of Food Safely Commission, Cabinet Office, 5-2-20 Akasaka, Minato-ku, Tokyo 107-6122, Japan.

## Pre-publication history

The pre-publication history for this paper can be accessed here:

http://www.biomedcentral.com/1471-2458/14/25/prepub
